# Using omics approaches to dissect the therapeutic effects of Chinese herbal medicines on gastrointestinal cancers

**DOI:** 10.3389/fphar.2022.884822

**Published:** 2022-09-23

**Authors:** Si-Yi Li, Wei-Jia Wang, Qiu-Yue Li, Peng-Hui Yang, Xin-Long Li, Yan Yan, Yong Yuan, Yi-Bin Feng, Ming Hong

**Affiliations:** ^1^ Joint Laboratory for Translational Cancer Research of Chinese Medicine of the Ministry of Education of the People’s Republic of China, Guangzhou, China; ^2^ International Institute for Translational Chinese Medicine, Guangzhou University of Chinese Medicine, Guangzhou, China; ^3^ Dongguan Institute of Guangzhou University of Chinese Medicine, Dongguan, China; ^4^ Institute of Advanced Diagnostic and Clinical Medicine, Zhongshan People’s Hospital, Affiliated Zhongshan Hospital of Sun Yat-sen University, Zhongshan, China; ^5^ Science and Technology Innovation Center, Guangzhou University of Chinese Medicine, Guangzhou, China; ^6^ Institute of Gastroenterology, Guangzhou University of Chinese Medicine, Guangzhou, China; ^7^ Guangzhou University of Chinese Medicine, Guangzhou, China; ^8^ School of Chinese Medicine, The University of Hong Kong, Hong Kong, China

**Keywords:** Chinese herbal medicines, omics, gastrointestinal cancers, side effects, review

## Abstract

Chinese herbal medicines offer a rich source of anti-cancer drugs. Differences between the pharmacology of Chinese herbal medicines and modern synthetic chemicals hinder the development of drugs derived from herbal products. To address this challenge, novel omics approaches including transcriptomics, proteomics, genomics, metabolomics, and microbiomics have been applied to dissect the pharmacological benefits of Chinese herbal medicines in cancer treatments. Numerous Chinese herbal medicines have shown potential anti-tumor effects on different gastrointestinal (GI) cancers while eliminating the side effects associated with conventional cancer therapies. The present study aimed to provide an overview of recent research focusing on Chinese herbal medicines in GI cancer treatment, based on omics approaches. This review also illustrates the potential utility of omics approaches in herbal-derived drug discovery. Omics approaches can precisely and efficiently reveal the key molecular targets and intracellular interaction networks of Chinese herbal medicines in GI cancer treatment. This study summarizes the application of different omics-based approaches in investigating the effects and mechanisms of Chinese herbal medicines in GI cancers. Future research directions are also proposed for this area of study.

## Introduction

Gastrointestinal (GI) cancer refers to malignant conditions of the gastrointestinal system and encompasses colorectal, gastric, hepatocellular, pancreatic, cholangiocarcinoma, and esophageal cancers (Torre and Bray et al., 2015; Abdelfatah et al., 2016). Most patients with GI cancers are diagnosed at advanced stages due to the limitations of diagnostic approaches and conventional therapy (Rahbari et al., 2016). It is of paramount importance to improve the early diagnosis and treatment efficacy, thereby reducing the mortality arising from GI cancers. For proper diagnosis and treatment of GI cancers, it is imperative to develop effective screening methods capable of detecting precancerous lesions and revealing the pathological and molecular mechanisms of GI cancers.

**TABLE 1:** Applications of genomics approaches for the testing of Chinese herbal medicines in GI cancers.

Cancer type	Method	Active ingredients	Chinese herbal medicines source	Main anti-GI cancer mechanism	Reference
Gastric cancer	Microarray	Ursolic acid	*Prunella vulgaris* L	Diminishing the proliferation and metastasis of gastric cancer *via* the regulation of Hippo pathway through Rassf1	Kim et al. (2019)
	Microarray	Periplocin	Cortex periplocae	Upregulated the expression of EGR1 and ERK1/2	Li et al. (2016)
	High-throughput RNA sequencing, real-time PCR	P-coumaric acid	Edible plants	Modulating the expression of certain miRNAs	Jang et al. (2020)
	Gene microarray assay	SAN	*Papaver somniferum* L	Inhibition of miR-96-5p and miR-29c-3p expressions, and subsequent activation of the MAPK/JNK signaling pathway	Dong et al. (2019)
	cDNA microarray assay	Quercetin	Common vegetables and fruits	Seven upregulated and 15 downregulated genes are associated with the apoptotic cell death	Shang et al. (2018)
	miRNA sequencing and RNA sequencing	Berberine	*Coptis chinensis*	Inhibit the proliferation of SGC-7901 cells and induce apoptosis	Yang et al. (2018)
	FuGENE 6 transfection reagent	Oleic acid	Olive oil	Inhibits Her-2/neu gene promoter activity through the action of PEA3 protein	Menendez et al. (2006)
	Quantitative reverse transcription-PCR arrays	PepE	*Peperomia dindygulensis*	Inhibits DNA methylation	Wanga et al. (2018)
HCC	cDNA microarray hybridization	*Perilla frutescens* leaf extracts	*Perilla frutescens* leaf	Increases the expression of apoptosis-related genes and apoptosis inducing in HepG2 cells	Lin et al. (2007)
	cDNA microarray	Curcumin	Turmeric or curry powder	Inhibited the expression of the PKC gene	Kao et al. (2011)
Esophageal cancer	Microarray analysis	Sulforaphene	Radish seeds	Decrease SCD and CDH3 expression and upregulate MAP2K3 and GADD45B expression	Han et al. (2020)
	RNA sequencing	Phlorizin	Sweet tea leaves	Upregulated 749 genes and downregulated 1,405 genes, and the autophagy marker gene, P62/SQSTM1 had high expression levels	Jia et al. (2021)
Pancreatic cancer	mRNA microarray	Fv1	*Fucus vesiculosus*	Upregulated the cell cycle inhibitor p57, and certain suppressed downstream targets that are inhibited by p57	Geisen et al. (2015)
	Microarray analysis	Paeoniflorin	*Paeonia* i Pall	Inhibited pancreatic cancer growth by upregulating HTRA3	Li et al. (2017)
Esophageal cancer	DNA microarray	bisPMB	Garlic	Regulating protein processing in the endoplasmic reticulum (ER) and the unfolded protein response	Siyo et al. (2017)

**TABLE 2:** Applications of proteomics approaches to study the mechanisms of Chinese herbal medicines in GI cancers.

Cancer type	Method	Active component	Chinese herbal medicine source	Main anti-GI cancer mechanism	Reference
HCC	2-DE and MALDI-TOF MS	*Scutellaria barbata* polysaccharides	*S. barbata*	Increased RNA-binding heterodimer (Srp9/14)	Li et al. (2019)
	iTRAQ combined with 2D-LC-MSMS	Viscum coloratum (Kom.)	Nakai	113 and 198 differentially expressed proteins were identified	Chai and Zhao, (2017)
	Two-dimensional difference gel electrophoresis and matrix-assisted laser desorption/ionization time-of-flight mass spectrometry	Platycodin D (PD)	Platycodonis Radix	Decreased RPS12, EMG1, and KRT1	Lu et al. (2015)
	Reversed-phase proteomic array analysis (RPPA)	Fraxini	Mistletoe	Protein levels of Bcl-xl, Bcl2, pRb and CDK1 were reduced, whereas cleaved caspase 7 were elevated	Yang et al. (2019)
Gastric cancer	Shotgun proteomic analysis	CHP	*Aspongopus chinensis* Dallas	*Via* suppression of cancer cell proliferation and acceleration of apoptosis	Tan et al. (2019)

**TABLE 3:** Application of metabolomics approaches to study Chinese herbal medicines in GI cancers.

Cancer type	Method	Active component	Chinese herbal medicines source	Main anti-GI cancer mechanism	Reference
HCC	1H-NMR spectroscopy assay	Ethyl acetate extract of *Crithmum maritimum*	*Crithmum maritimum*	Induced cytostasis was regulated *via* a multi-effects action, targeting pivotal metabolic processes in liver cancer cells	Gnocchi et al. (2021)
	GC–MS assay coupled with multivariate statistical analysis	Ethyl acetate extract of *Crithmum maritimum*	*Crithmum maritimum*	Suppress HCC cell proliferation	Kim et al. (2015)
	UPLC–MS	Betulinic acid	Forsythiae Fructus	Anti-cancer	Bao et al. (2018)
Colon cancer	UPLC–MS/MS assay	Shikonin	*Lithospermum* erythrorhizon	Inhibit CRC cells growth *via* various signaling pathways: glutathione metabolism, arginine biosynthesis, purine metabolism, beta-alanine metabolism, and arginine biosynthesis	Yang Chen et al. (2020)
	Multivariate data analysis	Ionic liquid-Graviola fruit pulp extract (IL-GPE)	Ionic liquid-Graviola fruit pulp	Selectively suppressing cancer cell proliferation and energy metabolism	Daddiouaissa et al. (2021)
	UPL−ESI−QTOFMS assay	Nutmeg extracts	nutmeg	Inhibit CRC development by alleviating metabolic disorders and modulating gut microbial metabolism	Li et al. (2015a)

**TABLE 4:** Applications of transcriptomics approaches to study Chinese herbal medicines in GI.

Cancer type	Method	Active component	Chinese herbal medicines source	Main anti-GI cancer mechanism	Reference
Gastric cancer	RNA-seq	Tanshinone IIA	*Salvia miltiorrhiza*	Inhibited the gastric cancer cell line AGS by suppressing the cancer cells’ glucose metabolism	Lin et al. (2015)
Colon cancer	RNA-seq	Apigenin	*Apium graveolens* L	Suppressed CRC cell proliferation by decreasing the expression of E2F1/3 and by regulating miRNA-205-4p	Cheng et al. (2021)
	RNA-seq	Baicalin	*Scutellaria baicalensis* Georgi	Suppress the malignant phenotypes of CRC *via* regulating circRNA MYH89/miR-761 axis	Lu et al. (2015)
	RNA-seq	*Thalassia testudinum* extracts	*Thalassia testudinum*	Trigger multiple stress signaling pathways that induced CRC cell apoptosis	Hernandez-Balmaseda and Guerra et al. (2021)
	RNA-seq	Shikonin	*Lithospermum erythrorhizon*	Regulation of purine metabolism	Chen et al. (2020a)

**TABLE 5:** Applications of microbiomics approaches to study Chinese herbal medicines in GI cancers.

Cancer type	Method	Active component	Chinese herbal medicines source	Main anti-GI cancer mechanism	Reference
Colon cancer	16S amplicon library, miSeq sequencing, and QIIME analysis of microbial gut ecology	Curcumin	Turmeric or curry powder	Restored the relative abundance of the Lactobacillales order in colitic Il102/2 and in AOM/Il102/2 mice	McFadden et al. (2015)
	miSeq sequencing, and QIIME analysis of microbial gut ecology	Chinese ginseng extracts	Chinese ginseng	Enteric microbiome population-shift recovery and dysbiosis restoration	Yang and Shan et al. (2020)
	16S rRNA gene sequence analysis of the gut microbiota in fecal samples and bioinformatics analysis	BPIS	Foxtail millet bran	Remodel the overall structure of the gut microbiota from tumor-bearing mice toward that of the normal counterparts	Yang and Shan et al. (2020)

The omics field is a combination of high-throughput and specialized biotechnological assays, equipment, and algorithms comprising transcriptomics, genomics, interactomics, proteomics, phenomics, and metabolomics. Omics disciplines can also include subdisciplines, which require further specialization in computational and analytical methods (Pirih and Kunej, 2017). For genomics analyses, technical approaches mainly include RNA transfection, microRNA (miRNA) transfection, and real-time PCR analysis. Transcriptomics analyses consist of RNA sequencing, DNA microarray, and expressed sequence tag (EST) technology. Proteome profiler antibody arrays and SWATH-MS are common methods used in proteomics while IN NMR, LC-MS, and UPLC-Q-TOF/MS are used for metabolomic analyses. As for microbiomics, high-throughput sequencing and mass spectrometry are common approaches. To date, novel experimental methods, including high-throughput technologies, have been utilized for collecting large medical omics datasets, which have proven to be invaluable for oncology research.

Natural products with medicinal components possess potential pharmaceutical benefits in the treatment of cancer. Chinese herbal medicines that can be used to treat GI cancers include three categories; botanical drugs, Chinese herbal formulations, and herbal medicine compounds. For example, the botanicals Scleromitrion diffusum and *Hericium erinaceus* are commonly utilized to treat gastrointestinal diseases, including gastric cancer; they exert their anti-cancer actions by inhibiting tumor angiogenesis, proliferation, and promoting apoptosis [Lu et al., 2019 (PMID: 32265701); Li F. et al., 2015 (PMID: 24631140)]. Chinese herbal medicines have been shown to improve the efficacy of conventional GI cancer treatment, alleviate adverse effects, and reverse drug resistance (Hu et al., 2016). In addition, traditional Chinese medicine (TCM) syndrome is a patient-centered clinical manifestation profile, which contributes to the individualized treatment of cancer (Ji et al., 2016). The characteristics and advantages of TCM in cancer treatment are based on syndrome differentiation [Jiang et al., 2020 (PMID: 22322251)]. Combined with syndrome differentiation, the application of omics has great clinical significance in TCM treatment of GI cancers (Zhang and Shang, 2011). However, systematic summaries on how omics assist in understanding the mechanisms of Chinese herbal medicines in GI cancer treatment remain scarce.

**FIGURE 1:**
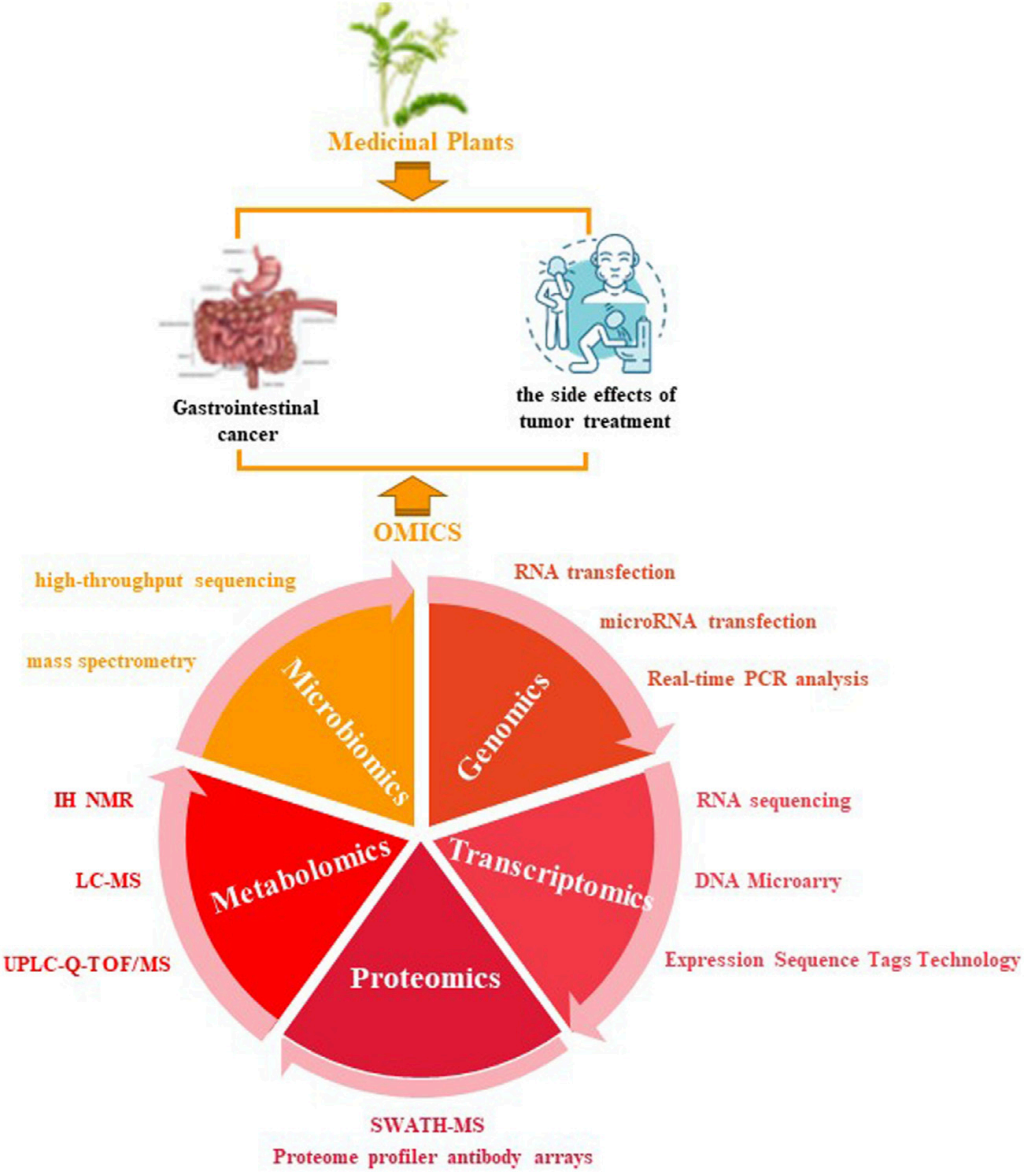
Application of omics assays in studying Chinese herbal medicines for treating gastrointestinal cancer. Application of omics approaches in GI cancer treatment with Chinese herbal medicines.

This study provides a comprehensive review of the latest omics approaches used for characterizing the anti-tumor actions of Chinese herbal medicines on GI cancers and their ability to attenuate adverse effects arising from conventional cancer therapy. We generalized several omics methods that reveal the potential molecular mechanisms of anti-tumor Chinese herbal medicines in intracellular signaling pathway networks, key targets, and host gut microbiota (Figure 1). Omics data can be subsequently analyzed and validated by systems biology, network pharmacology, *in vitro* and *in vivo* experiments, and ultimately in clinical trials. In addition, we also discuss existing limitations and prospects of omics approaches in this field.

Most clinical GI cancer cases include gastric cancer, colorectal cancer, hepatocellular carcinoma (HCC), esophageal cancer, pancreatic cancer, and cholangiocarcinoma (Torre et al., 2015; Abdelfatah et al., 2016). Omics approaches are widely utilized to explore the efficacy and molecular mechanisms of Chinese herbal medicines in treating GI cancers. Despite the reproducibility, noise disturbance, and complexity of omics, the utilization of omics approaches can improve the likelihood of identifying active anti-tumor ingredients from herbal medicines. Moreover, omics approaches can be used to further dissect the molecular mechanisms underlying the anti-tumor activity of Chinese herbal medicines, such as inducing apoptotic cell death, relieving oxidative damage, and regulating host immunity [Wang et al., 2014 (PMID: 25379508); Oyenihi et al., 2021 (PMID: 34770949)].

### Genomics

The field of genomics was initially used for DNA sequencing, from which it has rapidly expanded to more widely encompassing functional assessments—exploring the roles of both proteins and genes as well as their associated expression profiles. The emergence of novel herbal genomics research, together with advances in other omics approaches may improve the discovery of novel anti-GI agents arising from Chinese herbal medicines.

A previous study utilized genomics analysis to identify that PepE, a natural secolignan isolated from the Chinese botanical herb *Peperomia dindygulensis*, may prevent gastric cancer metastasis and inhibit DNA methylation in epigenetic cancer therapy (Wanga et al., 2018). RNA sequencing and miRNA sequencing found that berberine (BBR), isolated from *Coptis chinensis*, could inhibit the proliferation of SGC-7901 gastric cancer cells and induce cell apoptosis (Yang et al., 2018). In addition, through microarray experiments, ursolic acid, which exists in the whole grass of *Prunella vulgaris* L., the leaves of *Ilex rotunda* Thunb, and many other botanical drugs, was found to exhibit inhibitory effects on the metastasis and proliferation of gastric cancer cells by modulating the Hippo pathway *via* the tumor suppressor gene Rassf1 (Kim et al., 2019). Periplocin, an extract from the traditional herbal medicine cortex periplocae (*Periploca sepium* Bunge), may inhibit the proliferation of gastric cancer cells *via* the ERK1/2-EGR1 pathway (Li et al., 2016). Furthermore, a recent study used high-throughput RNA sequencing, real-time PCR, gene ontology (GO) enrichment, and the Kyoto Encyclopedia of Genes and Genomes (KEGG) pathway analyses, to show that p-coumaric acid, a phenolic ingredient isolated from various Chinese herbal plants, exhibits remarkable anti-tumor effects against the gastric cancer cell line SNU-16 by regulating miRNA expression (Jang et al., 2020). Furthermore, cetuximab and *β*-elemene, bioactive compounds isolated from the Chinese botanical drug Curcumae Rhizoma, can inhibit tumor growth and lymph node metastases in mice with colorectal cancer (Chen et al., 2020). *Tupistra chinensis* Baker (*Rohdea chinensis*), an anti-tumor Chinese botanical drug, inhibits the growth of gastric cancer *in vivo* (Wang et al., 2021). Ying et al. (2018) systematically illustrated the therapeutic potential of botanical drugs for esophageal cancer treatment *in vitro* and *in vivo*. The aforementioned studies indicate that Chinese herbal medicines and their active compounds may offer potential therapeutic value for the treatment of GI cancers by regulating cell apoptosis, proliferation, and metastasis.

Hepatocellular carcinoma (HCC) is an increasingly prevalent difficult-to-manage malignant liver disease. Omics approaches may reveal the anti-HCC mechanisms of various Chinese herbal medicines, such as inducing apoptotic cell death, autophagy, and regulating various non-coding RNAs (Dai et al., 2022). However, very few reports have utilized genomics experiments to investigate the anti-HCC effects of Chinese herbal medicines. In this field, cDNA microarrays comprise the most widely used genomics technology. A recent study that utilized a cDNA microarray assay found that curcumin could regulate various kinase gene expressions in HCC. Among the 79 kinase genes, the expressions of 31 genes were significantly increased while 29 genes were decreased, suggesting that curcumin might inhibit HCC cell proliferation by targeting the protein kinase C pathway (Kao et al., 2011). Using cDNA microarray hybridization, the *Perilla frutescens* extract (PLE) was shown to induce apoptosis by regulating the expression of numerous apoptosis-related genes in HepG2 cells (Lin et al., 2007). Although the aforementioned studies highlight the pivotal role of genomics approaches in discovering novel anti-HCC agents, there remain a considerable number of Chinese herbal medicines that await genomic investigations.

Several studies have shown that genomics could help characterize the anti-cancer mechanisms of numerous Chinese herbal medicines for esophageal cancer. For example, microarray quantification and real-time PCR showed that treatment with *Andrographis paniculate* (AP) resulted in decreased expression of AXIN2, WNT4, RTKN2, EGFR, OLFML1, BMP4, PDGFRB, SMAD7, MYH10, ABCA13, and ABCB1, while ZNF704 and ASNS were upregulated in esophageal cancer cells. These results indicated that several intracellular signaling processes associated with cell apoptosis, proliferation, metastatic processes, intercellular adhesion, and drug resistance were remarkably modulated after AP treatment.

A novel derivative of ajoene, BisPMB can be isolated from the Chinese herbal medicine garlic. In a recent study, a DNA microarray assay was applied in combination with gene ontology, KEGG, and an ingenuity pathway analysis to explore the active mechanisms of BisPMB in esophageal cancer. The results showed that BisPMB can inhibit esophageal cancer cell proliferation through the unfolded protein response activation *via* the GADD153/CHOP pathway (Siyo et al., 2017). Moreover, several studies have shown that microarray analysis could help reveal the underlying mechanism of Chinese herbal medicines in regulating esophageal cancer cell metastasis. Sulforaphene (SFE), a novel isothiocyanate in radish seeds, has been found to inhibit esophageal cancer cell metastasis and proliferation. Upon treating the esophageal cancer EC109 cell line with SFE, microarray analysis showed significant downregulation of the metastasis-related CDH3 and SCD mRNA, while p38 activators such as GADD45B and MAP2K3 were upregulated (Han et al., 2020). In addition, RNA sequencing technologies show that SQSTM1/p62 (autophagy markers) was upregulated in esophageal cancer cells after treatment with phlorizin, the main component of the sweet tea leaf (*Lithocarpus polystachyus* Rehd). These data suggest that phlorizin can inhibit autophagy in esophageal cancer by regulating the ubiquitin-binding protein SQSTM1/P62 (Jia et al., 2021).

In pancreatic cancer, microarray assays are the most frequently applied genomics technology for exploring potential anti-cancer mechanisms of Chinese herbal medicines. For example, by using microarray assays, Fv1, an extract from *Fucus vesiculosus* was shown to regulate the cell cycle inhibitor p57, thereby leading to cell cycle arrest in pancreatic cancer cells (Geisen et al., 2015). Another study found that paeoniflorin could reduce cell proliferation and induce apoptosis in pancreatic cancer cells. Through microarray analysis, HTRA3 was significantly increased in paeoniflorin-treated cells, further inhibiting cell proliferation and inducing cell apoptosis (Li et al., 2017). Green tea, made from *Camellia sinensis* leaves, has been used as both a beverage and medicine in traditional Chinese culture. A recent study used microarrays to show that tea extracts offer an effective preventative for the tumorigenesis of pancreatic cancer cells; the downregulation of ID1, which is highly expressed in cancer cells, may be related to these inhibitory effects (Zheng et al., 2019).

### Proteomics

Proteomics assays include shotgun proteomic analysis, two-dimensional liquid chromatography–tandem mass spectrometry (2D-LC–MSMS), isobaric tags for relative and absolute quantification (iTRAQ), matrix-assisted laser desorption/ionization time-of-flight mass spectrometry, and two-dimensional difference gel electrophoresis. The application of proteomics assays has offered preliminary biological evidence for the potential utility of Chinese herbal medicines in treating gastric cancer. For example, Tan et al. (2019)utilized a shotgun proteomic analysis and found that cytochrome c, an active component purified from Aspongopus chinensis, might be a noteworthy anti-cancer agent for gastric cancer treatment. The Weining granule (WNG), a Chinese medicine formula that comprises Poria cocos (Chinese name: Fuling, FL), *Astragalus mongholicus* Bunge (Chinese name: Huangqi, HQ), Fructus Lycii (Chinese name: Gouqizi, GQZ), Curcumae rhizoma (Chinese name: Ezhu, EZ), *Solanum nigrum* (Chinese name: Longkui, LK), Ranunculus ternatus Thunb. (Chinese name: Maozhuacao, MZC), and Rhizoma Paridis (Chinese name: Chonglou, CL) has been widely used for treatment of GI patients. Liang et al. (2021)showed that WNG treatment altered the abundance of 192 proteins in gastric cancer cells. These proteins were associated with carbon metabolism, cholesterol metabolism, alanine, glutamate and aspartate metabolism, and TNF signaling pathways in gastric cancer.

In HCC, the mechanisms of action for Chinese herbal medicines have been characterized using multiple proteomics approaches. Previous studies have shown that reduced actin-binding protein profilin 1 (PFN1) might be associated with HCC and can indicate a poor prognosis. Based on proteomic analyses, guttiferone K (GUTK) isolated from the botanical drug genus Garcinia, was shown to inhibit HCC cell invasion and migration by regulating PFN1 expression (Shen et al., 2016). Platycodin D (PD) is a triterpenoid saponin extracted from *Platycodon grandiflorus*. After PD-treatment in HCC cells, 16 proteins were increased and three proteins were decreased, as determined by matrix-assisted laser desorption/ionization time-of-flight mass spectrometry and two-dimensional difference gel electrophoresis (Lu et al., 2015). Recently, a study using 2D-LC–MSMS combined with an iTRAQ assay identified 198 and 113 differentially expressed proteins after Viscum coloratum polysaccharide 2 (VCP2) treatment in HCC and normal hepatic cells, respectively. These findings broaden our understanding of the anti-tumor mechanisms of the active ingredients in Viscum coloratum and offer novel assays for screening proteins, which comprise potential targets of polysaccharides (Chai and Zhao, 2017). In addition, a reversed-phase proteomic array (RPPA) analysis combined with proteomics found that fraxini, a mistletoe extract, suppresses the proliferation of HCC by decreasing the expression of c-Myc [Yang et al., 2019 (PMID: 31015523)]. In 2019, Hou et al. (2020) used proteomic techniques to elucidate the anti-HCC mechanism of dihydroartemisinin, a semi-synthetic derivative of artemisinin. They showed that dihydroartemisinin can up-regulate the APOA1 protein and downregulate GALNT10. Huacheng et al. analyzed the protein expression profiles of human HCC cells after treatment with Gexia Zhuyu Decoction (GXZY). Protein electrophoretic profiles of the treatment and control groups were analyzed by mass spectrometry, and six metastasis-related proteins were identified, suggesting that GXZY drug-containing sera may alter protein expression in human HCC cells (Hua-cheng and Jian-gang, 2015).

In colon cancer, proteomics assays have also been extensively used to interrogate the underlying mechanisms of Chinese herbal medicines. Xiong et al. (2020)explored the anti-tumor efficacy of AUCAN, a type of dibenzofuran, *in vitro* and *in vivo* colorectal cancer (CRC) models. Further mechanistic studies using proteomic and functional clustering analyses showed that AUCAN treatment upregulated 24 proteins and downregulated 42 proteins, suggesting that AUCAN treatment may regulate multiple genes in CRC. Chen et al. (2021)conducted a label-free based quantitative proteomic analysis to evaluate the protein expression profiles in colon cancer cells treated with the traditional Chinese medicine *Camellia nitidissima* Chi (CNC). They showed that CNC regulated CRC cell proliferation *via* regulation of ferroptosis signaling. The presented evidence indicates that proteomics analyses can be used to interrogate the mechanisms of Chinese herbal medicines in GI cancer treatment.

### Metabolomics

Metabolomics approaches primarily consist of ultra-performance liquid chromatography–tandem mass spectrometry (UPLC–MS/MS), metabolic profiling, UPL−ESI−QTOFMS, 1H-NMR spectroscopy, and GC–MS combined with multivariate statistical analyses. Previously, metabolomics was used to explore the anti-gastric cancer mechanisms of Chinese herbal medicines. Several studies have utilized proteomics in the study of Chinese herbal medicine in HCC and colon cancer. Metabolomics has the potential to reveal the active anti-cancer components present in Chinese herbal medicines.

Based on 1H-NMR spectroscopy, Gnocchi et al. (2021)found that the ethyl acetate extract of *Crithmum maritimum* can reverse the Warburg effect (preferential use of glycolysis rather than oxidative phosphorylation for energy production by tumor cells) in HCC cells by decreasing intracellular lactate. The ethyl acetate extract can also inhibit protein anabolism by reducing the intracellular level of amino acids and alter membrane biosynthesis by lowering choline and phosphocholine contents in HCC. Furthermore, Kim et al. (2015) applied a GC–MS assay coupled with multivariate statistical analysis and described the multitarget action of the ethyl acetate extract of *Crithmum maritimum* in suppressing HCC cell proliferation. A metabolomics investigation utilizing LC–MS tested the active components of toad venom that show potential anti-tumor mechanisms in HCC. They showed that the combination of toad venom and a Chinese toad-shortening base could cause HCC tumor metabolic disorders, and reduce mitochondrial membrane potential, relieve oxidative damage of cancer cells, and inhibit energy metabolism. Metabolomics analysis found that terpenoids, flavonoids, alkaloids, phenylpropanoids, and fatty acids are the most abundant bioactive components of *Astragalus membranaceus*, with anti-tumor, anti-oxidation, and anti-inflammation effects (Wu et al., 2020). Based on UPLC–MS-based metabolomics approaches, betulinic acid was identified as the most active anti-cancer compound in Forsythiae Fructus (Bao et al., 2018).

In colon cancer, metabolomics have revealed the mechanisms underlying novel cancer treatments using Chinese herbal medicines. Yang et al. used an UPLC–MS/MS assay to identify that shikonin could inhibit CRC cell growth *via* various signaling pathways that included glutathione metabolism, arginine biosynthesis, purine metabolism, beta-alanine metabolism, and arginine biosynthesis (Chen et al., 2020b). Daddiouaissa et al. (2021)performed metabolic profiling and utilized multivariate data analysis combined with an ingenuity pathway analysis (IPA) to explore the effects of treating CRC cells with the ionic liquid-Graviola fruit pulp extract (IL-GPE). A pathway analysis of metabolomic profiles showed significant alterations in pathways related to cancer cell growth and energy metabolism, including aerobic glycolysis, amino acid metabolism, urea cycle, and ketone body metabolism [Pan et al., 2021 (PMID: 34547237)]. The authors concluded that IL-GPE might be useful for the treatment of CRC *via* selectively suppressing cancer cell proliferation and energy metabolism (Daddiouaissa et al., 2021). Li et al. adopted an UPL−ESI−QTOFMS assay to examine the metabolic signatures of CRC cells after nutmeg treatment, demonstrating inhibition of CRC development due to alleviation of dysregulated lipid metabolism and normalization of gut microbial metabolism (Li W. B. et al., 2015). Dysfunction of the gut microbiome contributes to the development of GI cancers [Peng et al., 2020 (PMID: 32855157); Song et al., 2020 (PMID: 31586566)]. Chinese herbal medicines have shown considerable utility in the treatment of metabolic disorders *via* their regulation of the gut microbiota. Zhang et al. reported that Chinese herbal medicines can affect glucose and lipid metabolism by modulating the gut microbiota, including anti-inflammatory bacteria, lipopolysaccharide- and short-chain fatty acid-producing bacteria, and bacteria with bile-salt hydrolase activity [Zhang et al., 2020 (PMID: 33197760)]. Based on metabolomics approaches, Chinese herbal medicines might provide a promising complementary therapy for CRC patients.

### Transcriptomics

Transcriptomics comprises high-throughput assays to assemble a complete set of RNA transcripts in a specific cell or culture of cells under specific conditions. Because of their accuracy, sensitivity, high throughput, reproducibility, and specificity, DNA microarrays have been widely applied to study the transcriptomics of Chinese herbal medicine. In addition, RNA sequencing (RNA-seq) is also widely used and can be applied to the analysis of the therapeutic mechanisms of Chinese herbal medicines [Chambers et al., 2018 (PMID: 30264869); Duan et al., 2021 (PMID: 34074345)]. Therefore, in this review, we focus on an overview of transcriptomics assays in GI cancer treatment using Chinese herbal medicines.

Tanshinone IIA (TIIA) is a diterpene quinone extract isolated from the Chinese botanical drug *Salvia miltiorrhiza*. In gastric cancer, the TIIA anti-tumor effects and mechanisms were analyzed by RNA-seq transcriptomics combined with iTRAQ. TIIA significantly inhibited the gastric cancer cell line AGS by controlling the glucose metabolism of cancer cells (Lin et al., 2015).

Transcriptomic analysis can interrogate various nucleic acids including micro RNAs (miRNA), long non-coding RNAs (lncRNAs), and circular RNAs (circRNAs), to explain the therapeutic effects of herbal medicines on cancers. Lan et al. (2021)investigated the transcriptome-level mechanisms of baicalin-mediated anti-tumor effects on osteosarcoma (OS) and identified 58 lncRNAs and 31 miRNAs that responded to the treatment. In addition, baicalin can suppress the malignant phenotypes of CRC by regulating the circRNA MYH89/miR-761 axis (Zhang et al., 2021). Based on high-throughput sequencing, Cheng et al. (2021) found that the herbal ingredient apigenin (API) suppressed CRC cell proliferation by decreasing E2F1/3 and regulating miRNA-215-5p. However, to our knowledge, the potential involvement of GI cancer circRNA modulation by Chinese herbal medicines has not been interrogated.

Based on transcriptome profiling of colon cancer cells after treatment with *Thalassia testudinum* extracts (TTE), a recent study found that TTE can trigger multiple stress signaling pathways (endoplasmic reticulum stress, unfolded protein stress, DNA damage, and nitrosative stress) that induced CRC cell apoptosis. This evidence supports the potential use of TTE in CRC treatment (Hernandez-Balmaseda and Guerra et al., 2021). Shikonin is a commonly used naphthoquinone ingredient isolated from the Chinese botanical drug *Lithospermum erythrorhizon*. . Chen et al. (2020c) combined metabolomics and transcriptomics to assess the anti-cancer activity of shikonin in human CRC cells. They showed that shikonin exhibits significant anti-tumor activity and that the regulation of purine metabolism may contribute to the anti-cancer effects of shikonin.

### Microbiomics

Both environmental and host factors can contribute to the development of GI cancers. The intestinal microbiota (IM) can directly affect health and play a pivotal role in GI cancer development. In recent years, with the rapid development of high-throughput sequencing technology, the relationship between the IM and their metabolites, and the development, diagnosis, and treatment of CRC have been elucidated. Herein, we review previous work utilizing microbiomics to explore the potential mechanisms of Chinese herbal medicine extracts for the treatment of gut microbiota-related GI cancers.

Curcumin, derived from the rhizome of the Curcuma longa plant, was found to function as an analgesic, antiseptic, antioxidant, anti-inflammatory, chemo-sensitizing, chemo-preventive, and radio-sensitizing agent on colon tumorigenesis (McFadden et al., 2015). This work was completed using a 16S amplicon library, miSeq sequencing, and quantitative insights into microbial ecology (QIIME) analysis of the gut. The role of Chinese ginseng in modulating the intestinal microbiome for colon cancer prevention has also been explored through microbiomics. It was suggested that the CRC chemo-preventive effects of Chinese ginseng are mediated through enteric microbiome population-shift recovery and dysbiosis restoration. Furthermore, ginseng regulation of microbiome balance contributes to the maintenance of enteric homeostasis (Wang et al., 2018). The effects of extracts from Chinese herbal medicines in treating colitis-associated carcinogenesis have been explored using 16S rRNA gene sequence analysis and bioinformatic analysis of the gut microbiota *via* examinations of fecal samples. Bound polyphenol of inner shell (BPIS) is extracted from foxtail millet bran, and was shown to remodel the overall gut microbiota composition toward normal in tumor-bearing mice; this included two phyla and eight genera, together with modulations of various genes that regulate 17 signal pathways in colon cancer (Yang et al., 2020). Sui et al. (2020)demonstrated the inhibitory effects of the traditional Chinese botanical drug (Yi-Yi-Fu-Zi-Bai-Jiang-San) on colon carcinogenesis by examining gut bacterial diversity and performing human-to-mouse fecal microbiota transplantation experiments. Jiang et al. (2020)utilized 16S rDNA sequencing of intestinal flora and discovered that Wu Mei Wan, a nine-ingredient Chinese Medicine formula, can attenuate colitis-associated CRC by balancing tumor-promoting and tumor-suppressing bacteria. Alisol B 23-acetate, a natural tetracyclic triterpenoid found in Alismatis rhizome, also possesses therapeutic effects on colitis-associated cancers *via* a reduction of pathogenic bacteria, such as *Klebsiella*, *Citrobacter*, and *Akkermansia* (Zhu et al., 2021).

### Omics facilitates the understanding of mechanisms of Chinese herbal medicines in treating CI cancers and alleviating the adverse effects of conventional therapies

Chinese herbal medicines can effectively alleviate or even eradicate the adverse effects of conventional tumor treatments. Pain, bloating, nausea, vomiting, and diarrhea are common adverse effects of radiation exposure or chemotherapy in patients with GI cancers. Based on omics approaches, we can characterize the potential mechanisms of Chinese herbal medicines in alleviating these adverse effects. The therapeutic effects and mechanisms of Chinese herbal medicines on GI cancers can be explored by genomics analysis. For example, DNA microarray was applied to analyze the gene expression profiles of HCC cells treated with San-Huang-Xie-Xin-Tang (SHXXT), thereby obtaining the therapeutic mechanisms of SHXXT on HCC [Lin et al., 1994 (PMID: 7898124)]. Metabolomics is an analytical profiling technique for measuring and comparing metabolites in biological samples. Combining high-throughput analytical chemistry and multivariate data analysis, metabolomics provides an important foundation for metabolic mechanisms of Chinese herbal medicines treating GI cancers. A recent study revealed the potential mechanisms of the anti-inflammatory effects of Glycyrrhiza extracts in chemotherapy-induced colitis *via* a plasma metabolomics approach. Glycyrrhiza Linn., also known as licorice, is a commonly used Chinese herbal medicine. Glycyrrhiza contains multiple flavonoids and exhibits significant anti-inflammatory effects both *in vitro* and *in vivo* [Leite et al., 2022 (PMID: 35456938)]. Eleven differential endogenous metabolites associated with the anti-inflammatory effects of licorice flavonoids were shown, such as linoleic acid, sphingosine, corticosterone, leukotriene B4, and tryptophanamide. The multi-pathway-integrated mechanisms of licorice flavonoid action may alleviate chemotherapy-induced colitis in GI patients, which can improve the development of novel complementary agents for reducing the adverse effects of conventional GI treatment (Yang et al., 2020). Previous studies have suggested that various chemotherapy agents must be weighed against possible adverse effects on the hepatic tissue in patients with GI cancers [You et al., 2017 (PMID: 28458594); Wang et al., 2020 (PMID: 18458704)]. For patients with pre-existing hepatic disorders, conventional cancer treatment carries a significant risk of hepatotoxicity which can lead to hepatitis, bleeding, or cholestasis. A recent study found that injection with the compound kushen can effectively rebalance the Smad7/TGF-β signaling pathway in HSCs and alleviate chemotherapy-induced hepatotoxicity in patients with GI cancers (Yang et al., 2020). In addition, oral mucositis (OM), which can interrupt conventional cancer treatment, can affect the control of the disease and quality of life in patients. Transcriptomics and metabolomics assays showed that the Chinese herbal Shuanghua Baihe tablet (SBT) can alleviate chemotherapy-induced OM in colon cancer patients by regulating linoleic acid metabolism, glycerophospholipid metabolism, and amino acid metabolism, as well as by inhibiting IL-17/TNF signal transduction to restore Treg and Th17 cell homeostasis in OM rats (Geng et al., 2021).

Based on these data, we offer that different omics can be jointly applied to explore the mechanisms of Chinese herbal medicines in treating GI cancers. Genomics, transcriptomics, and proteomics analyze the biological functions and molecular mechanisms at the DNA, RNA, and protein levels, respectively. Metabolomics can be used to quantify small molecules and metabolites in cells and biological systems. It provides a measure of the inputs and outputs of biological pathways and is considered to be particularly powerful in describing the functional cell state, in comparison to other omics approaches such as genomics and proteomics. In addition, microbiomics collectively characterizes and quantifies molecules responsible for the structure, function, and dynamics of a microbial community, contributing to understanding microbial behaviors under different environmental conditions and providing a more suitable environment for disease treatment. To properly and comprehensively understand the mechanisms of Chinese herbal medicines underlying their effects on GI cancers, all omics can be used to analyze cancer cells and tissues after treatment with Chinese herbal medicines. As a result, data that describe the biological functions, molecular mechanisms, and microbial alterations can be collected more comprehensively, thereby facilitating the development of more appropriate therapeutic strategies and clinical decision making. The application of multi-omics approaches can also be used to reduce adverse effects. A comprehensive application of all omics approaches enriches our understanding of the biological functions, molecular mechanism, and microbial alterations that take place in response to Chinese herbal medicine treatment of GI cancers.

## Conclusion and future prospects

Active components extracted from Chinese herbal medicines and their active ingredients are highly effective for treating GI malignancies. For example, berberine represses human gastric cell growth by inducing cytostatic autophagy (Zhang et al., 2020). In addition, Jianpi Yangzheng Xiaozheng Decoction can prevent the progress of gastric cancer and inhibit the gastric cancer epithelial–mesenchymal transition (Wu et al., 2019). Betulinic acid delays tumor growth and inhibits pulmonary metastasis (Chen et al., 2020b). Curcumin can suppress cell proliferation and induce apoptosis in gastric cancer cells (Sun et al., 2019). Yiqi Jianpi Huaji Decoction can inhibit gastric cancer cell proliferation, induce apoptosis, and increase sensitivity to chemotherapeutic agents by decreasing the expression of TUBB3, MRP, P-gp, and STMN1 (Li F. et al., 2015). Wang et al. (2020) showed, in clinical studies, that Banxia XieXin Decoction treatment is safe and effective for patients with advanced HCC). Moreover, in a study by Zhang et al. (2017), combined astragaloside IV and curcumin therapy inhibited tumor growth and angiogenesis in an orthotopic nude-mouse model of human hepatocellular carcinoma (Zhang et al., 2017). Baicalin, the major active ingredient of the Chinese herbal medicine *Scutellaria baicalensis*, induces colon cancer cell apoptosis by inhibiting miRNAs (Tao et al., 2018). In terms of pancreatic cancer, Astragalus polysaccharide enhanced the anti-tumor effects of apatinib by downregulating the phosphorylation of AKT, ERK, and MMP-9 (Wu et al., 2018). However, many Chinese herbal medicines have not been approved in clinical trials because of their incompletely characterized active components and molecular mechanisms. Thus, the application of various omics assays may improve the rapid development of novel anti-cancer agents from Chinese herbal medicines. In the current review, the aforementioned studies have indicated that multiple omics approaches can assist in validating potential therapeutic drugs and promote the understanding of the underlying mechanisms of Chinese herbal medicines in GI cancer treatment.

**FIGURE 2:**
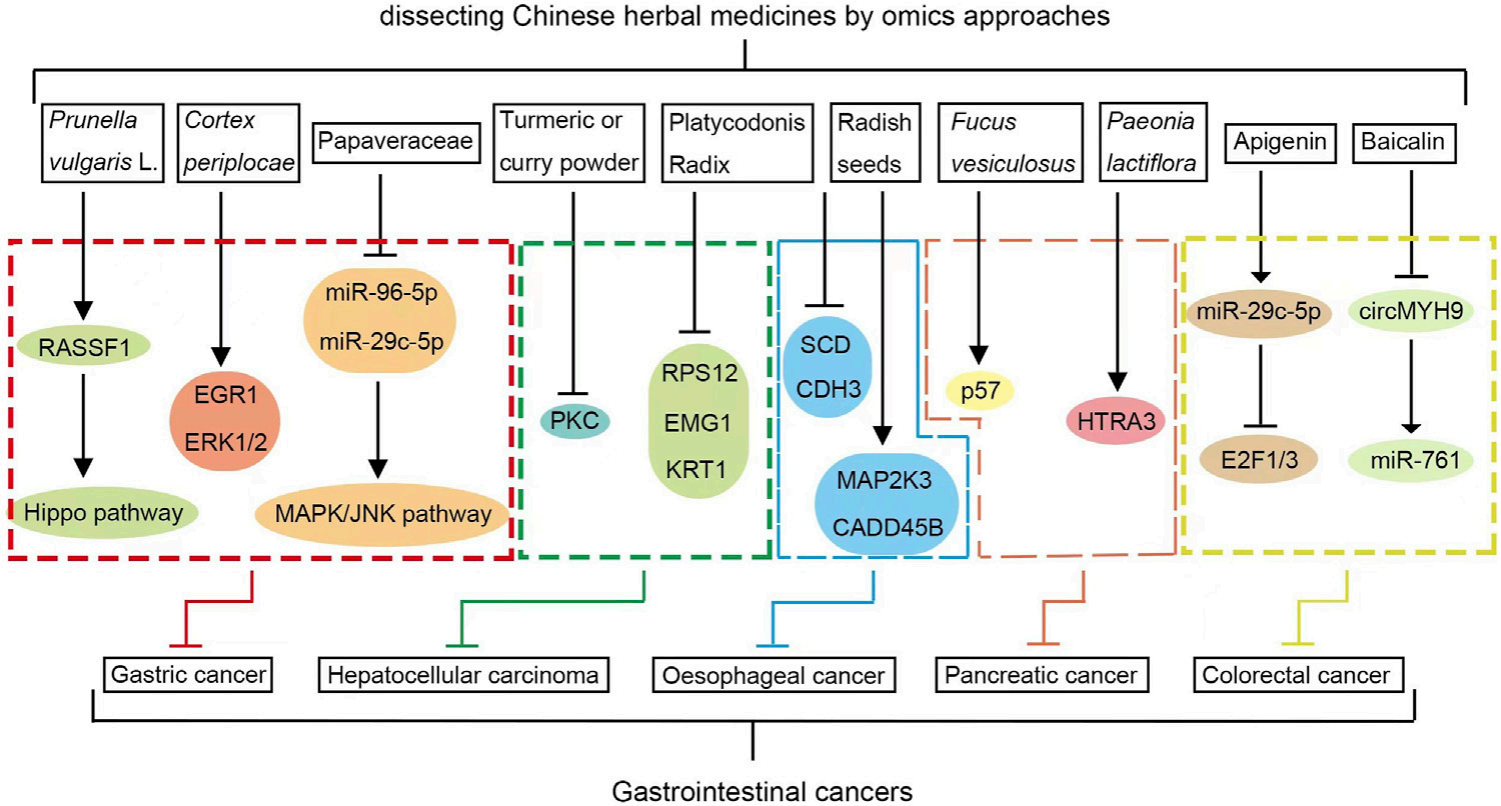
Underlying molecular mechanisms of Chinese herbal medicines in GI cancer treatment revealed by omics approaches.

Genomics, proteomics, metabolomics, transcriptomics, and microbiomics assays have been widely applied to analyze the role of Chinese herbal medicines in GI cancer treatment and its molecular mechanisms (Figure 2). However, many implementational limitations still exist. First, the complex ingredient list of Chinese herbal medicines hampers the process of characterizing bioactive ingredients and the initial investigation of their systemic actions. Second, we have not yet been able to deliver a comprehensive understanding of the toxicological effects and pharmacological activities associated with Chinese herbal medicines. Finally, the current chemical databases and laboratory equipment do not allow scientists to research as many of the chemical ingredients as possible, which poses further challenges when characterizing the mechanism as a whole.

In general, we suggest that additional bioinformatics analyses are integrated with omics assays to explore and exploit the inherently complex Chinese herbal medicines for GI cancers. Network pharmacology may effectively improve the development of current omics-based studies by directing treatment protocols based on the underlying biology of tumors. By combining omics assays with network pharmacological studies, future research can effectively incorporate the complex mechanisms of Chinese herbal medicine systems to generate biological networks and simplify the associated disease models. For example, through the integrative analysis of network pharmacology and RNA sequencing, Bushen-Jianpi-Jiedu Decoction combined with oxaliplatin can prolong postoperative CRC patient survival and improve their well-being; this is likely to involve the regulation of multiple signaling pathways (Yang et al., 2021). In addition, numerous public databases provide an unprecedented opportunity to dissect Chinese herbal medicines *via* bioinformatics analysis. The Traditional Chinese Medicine Information Database (TCMID) and Herb Ingredients’ Targets (HIT) database provide information about Chinese herbs, including formulas, herbal ingredients, therapeutic effects, and clinical indications [Ji et al., 2016 (PMID: 16376038); Yan et al., 2022 (PMID: 34986599)]. The Traditional Chinese Medicine Integrated database (TCMID) displays a network for integrative relationships between herbs and diseases, as well as active ingredients and targets [Huang et al., 2018 (PMID: 29106634)]. TCMID facilitates the study of combination therapy and understanding of the underlying mechanisms for Chinese herbal medicines at the molecular level. Combined with a bioinformatics analysis of public databases and omics, the biological functions and molecular mechanisms of Chinese herbal medicines in the treatment of GI cancers can be identified more accurately. Based on data from omics and public databases, Wu et al. screened out key botanical drugs and targets of Chinese herbal medicines for ulcerative colitis treatment by network pharmacology and enrichment analysis [Wu et al., 2020 (PMID: 32627503)]. By omics and bioinformatics analysis, systematic evaluation of Chinese herbal medicine for the treatment of GI cancers provides a basis for clinical prevention and treatment. Biological data generated by omics assays can be combined with systems biology to reveal the pharmacological substances, action targets, and mechanisms of Chinese herbal medicines (Cai et al., 2018). Thus, the combination of omics approaches and systems biology may contribute to the exploration of Chinese herbal medicines for treating GI cancers.
